# The excretory/secretory products of fifth-stage larval *Angiostrongylus cantonensis* induces autophagy via the Sonic hedgehog pathway in mouse brain astrocytes

**DOI:** 10.1371/journal.pntd.0008290

**Published:** 2020-06-01

**Authors:** Kuang-Yao Chen, Chien-Ju Cheng, Chih-Chieh Cheng, Kai-Yuan Jhan, Yi-Ju Chen, Lian-Chen Wang

**Affiliations:** 1 Department of Parasitology, School of Medicine, China Medical University, Taichung, Taiwan; 2 Department of Parasitology, College of Medicine, Chang Gung University, Taoyuan, Taiwan; 3 Graduate Institute of Biomedical Sciences, College of Medicine, Chang Gung University, Taoyuan, Taiwan; 4 Molecular Infectious Disease Research Center, Chang Gung Memorial Hospital, Taoyuan, Taiwan; PUCRS, BRAZIL

## Abstract

Angiostrongyliasis is induced by the nematode *Angiostrongylus cantonensis* and leads to eosinophilic meningitis and meningoencephalitis in humans. Excretory-secretory products (ESPs) are important investigation targets for studying the relationship between hosts and nematodes. These products assist worms in penetrating the blood-brain barrier and avoiding the host immune response. Autophagy is a catabolic process that is responsible for digesting cytoplasmic organelles, proteins, and lipids and removing them through lysosomes. This process is essential to cell survival and homeostasis during nutritional deficiency, cell injury and stress. In this study, we investigated autophagy induction upon treatment with the ESPs of the fifth-stage larvae (L5) of *A*. *cantonensis* and observed the relationship between autophagy and the Shh pathway. First, the results showed that *A*. *cantonensis* infection induced blood-brain barrier dysfunction and pathological changes in the brain. Moreover, *A*. *cantonensis* L5 ESPs stimulated autophagosome formation and the expression of autophagy molecules, such as LC3B, Beclin, and p62. The data showed that upon ESPs treatment, rapamycin elevated cell viability through the activation of the autophagy mechanism in astrocytes. Finally, we found that ESPs induced the activation of the Sonic hedgehog (Shh) signaling pathway and that the expression of autophagy molecules was increased through the Shh signaling pathway. Collectively, these results suggest that *A*. *cantonensis* L5 ESPs stimulate autophagy through the Shh signaling pathway and that autophagy has a protective effect in astrocytes.

## Author summary

In helminthes, Excretory-secretory products (ESPs) contains a wide range of molecules, including proteins, lipids, glycans, and nucleic acids, that assist in the penetration of host defensive barriers, reduction of oxidative stress, and avoid the host immune attack. It has been known as a key factor for parasite development, including feeding, invasion and molting. Therefore, ESPs is a valuable target for the investigation of the host-parasite relationships. However, only a few researches about the function of *Angiostrongyliasis cantonensis* ESPs have been verified to date. *Angiostrongyliasis cantonensis*, a blood-feeding nematode, and it is an important causative agent of eosinophilic meningitis and meningoencephalitis in human. Recent our studies have demonstrated that the *A*. *cantonensis* ESPs can induce oxidative stress, apoptosis, and immune response. In this study, we will use a mouse astrocytes as a model to investigate the signaling mechanisms of autophagy induction by ESPs treatment. First, the Microarray, Western blotting, and Transmission electron microscopy data demonstrated that *A*. *cantonensis* ESPs can induce autophagy generation in astrocytes. Next, ESPs-induced autophagy was activated via Sonic hedgehog (Shh) signaling, and it has a protective potential for astrocytes. These finding will provide new insights into the mechanisms and effects of the *A*. *cantonensis* ESPs.

## Introduction

*Angiostrongylus cantonensis*, a zoonotic parasitic nematode, is a major etiologic agent of cerebral angiostrongyliasis, including eosinophilic meningitis and eosinophilic meningoencephalitis [1,2,3]. The third-stage larvae (L3) infect nonpermissive hosts (humans) and only develop into the fifth-stage larvae (L5) in the CNS. L5 can still induce mechanical damage and host immune responses in the brain [4,5].

The secreted protein Hedgehog (Hh) and its pathway play important roles in animal development and the morphogenesis of a variety of tissues [6]. In humans, Hh has three homologs, namely, Sonic hedgehog (Shh), Desert hedgehog (Dhh) and Indian hedgehog (Ihh) [7]. Moreover, Shh deficiency can lead to neural, limb growth or foregut defects and induce many neurological diseases [8]. Shh signaling involves a series of inhibitory steps and triggers other common signaling pathways. In the absence of Shh, the transmembrane protein Patched (Ptc) inhibits Smoothened (Smo) function. When Shh protein is secreted, it can activate Smo by inhibiting Ptc function. Finally, Smo activates the transcription factor glioma-associated oncogene (Gli) family of transcription factors (Gli1–Gli3). Gli translocates to the nucleus and then regulates the expression of target genes involved in cell growth, survival, and proliferation [9].

Autophagy, a catabolic process that is responsible for digesting cytoplasmic organelles (the Golgi complex, mitochondria, and the endoplasmic reticulum), proteins, and lipids and then removing them through lysosomes in cells. This process is essential for cell survival and homeostasis during stress, such as nutritional deficiency, cell injury and oxidative stress [10–17].

Comprehensive analysis has demonstrated that autophagy trigger is appeared for microbial infection, such as bacteria, viruses, protozoa, and parasitic helminths [18–25]. In parasitic helminths, recent studies have revealed that the inhibition of autophagy or excess autophagy can induce cell apoptosis in *Echinococcus granulosus*. It can provide appropriate targets for new chemotherapeutic drugs for parasitic helminths [26].

During autophagy activation, a double layer membrane encircle vesicles and autophagosomes envelop intracellular microorganisms and then deliver them to lysosomes. Autophagosomes fuse with lysosomes to form autolysosomes for degradation [27]. The autophagic process is regulated by a group of autophagy-related proteins (Atgs). These genes were originally identified in yeast, and then many orthologs were identified in mammals [28]. Autophagy generation is regulated by the PI3K-Akt-mTOR signaling pathway [29–31]. First, the serine/threonine kinases UNC-51-like kinase -1 and -2 (ULK1 and ULK2) form a complex with Atg13 and FIP200, and then ULK1 triggers the activation of the Beclin1-Vps34-p150-Atg14-like (Atg14L) protein complex. Next, microtubule-associated protein 1A/1B-light chain 3 (LC3) and Atg12 play important roles in autophagosome formation and maturation. The lipophilic form of LC3 (LC3-II) is formed by LC3-I and phosphatidylethanolamine (PE) and is then steadily inserted into the autophagic membrane. On the other hand, the Atg12-Atg5-Atg16L complex is formed and leads to autophagic membrane elongation.

Our previous studies showed that the ESPs of *A*. *cantonensis* L5 may induce endoplasmic reticulum (ER) stress, oxidative stress and cell apoptosis in astrocytes. However, oxidative stress and cell apoptosis are reduced after Shh signaling pathway activation [1,32]. NF-κB can stimulate cytokine secretion through the Shh signaling pathway in *A*. *cantonensis* ESPs-treated astrocytes [9]. Therefore, Shh signaling plays an important role in *A*. *cantonensis* infection.

This project was designed to determine the relationship between autophagy and the Shh pathway upon *A*. *cantonensis* ESPs treatment. We found that upon ESPs treatment the number of autophagosomes in astrocytes is increased and that the Shh signaling pathway can protect astrocytes through autophagy activity.

## Materials and methods

### Ethics statement

All animal procedures in this study were approved by the Chang Gung University Institutional Animal Care and Use Committee (IACUC) in Taiwan (CGU107-086) and followed the guideline for Laboratory Animal Facilities and Care (The Council of Agriculture. Executive Yuan, ROC). Rats and mice were housed in plastic cages and provided with food and water ad libitum. The experimental animals were sacrificed by anesthesia with isoflurane (1 ml/min).

### Parasite and experimental infection

In this study, a Taiwan strain of *A*. *cantonensis* was employed and maintained in our laboratory. *Biomphalaria glabrata* snails and Sprague-Dawley (SD) rats were used to establish the life cycle [1]. The SD rats and BALB/c (H-2d) mice (8 weeks old) were purchased from the National Laboratory Animal Center (Taipei, Taiwan) or BioLASCO Taiwan Co., Ltd. (Taipei, Taiwan). On day 21 postinfection, the third-stage larvae (L3) of *A*. *cantonensi*s were isolated from the infected snails by digestion with 0.6% (w/v) pepsin-HCl (pH 2–3) for 1 h. Each BALB/c mouse was inoculated with 25 L3 via stomach intubation.

### Preparation and concentration of *A*. *cantonensis* excretory/secretory products

We used 200 *A*. *cantonensis* L3 to infect each rats, and brains were collected after anaesthetizing with 3% (v/v) isoflurane on day 21 post infection [1]. The lived *A*. *cantonensis* L5 were collected from the brain tissues and then removed tissue debris carefully by the dissecting microscope. They were washed with saline, phosphate-buffered saline (PBS), distilled water and RPMI containing a high concentration of antimycotic solution (200 units/ml penicillin G, 200 μg/ml streptomycin sulfate and 0.5 mg/ml amphotericin B) (Sigma-Aldrich, St. Louis, USA) before incubation in RPMI without fetal bovine serum (FBS) for 24, 48 and 72 h (37°C; 5% CO2). *A*. *cantonensis* L5 excretory/secretory products (ESPs) were collected and concentrated by Amicon Ultra-15 10K centrifugal filter devices (Merck Millipore, Germany). The concentration of ESPs from *A*. *cantonensis* L5 was detected with the Bio-Rad Protein Assay Kit (Bio-Rad, Hercules, CA, USA) according to the manufacturer’s instructions. These concentrated ESPs were employed to treat astrocytes, and cell morphology and protein expression level changes were detected [33].

### Mouse brain astrocyte culture

Cells from a mouse brain astrocyte cell line (CRL-2535) were purchased from American Type Culture Collection (ATCC) and employed in this study. Cells were cultured in Dulbecco’s modified Eagle’s medium (Corning, USA) containing 10% FBS. These cells were seeded on poly-L-lysine-coated culture plates or flasks and cultured at 37°C under 5% CO_2_. Based on GFAP staining, over 95% of the cultured cells were identified as astrocytes. When ESPs, activators, or inhibitors were used, the cells were incubated in serum-free DMEM for 24 h. Finally, the cells were pretreated with rapamycin (Sigma-Aldrich), chloroquine diphosphate (CQ) (Sigma-Aldrich), 3-methyladenine (3-MA) (Sigma-Aldrich), bafilomycin A1 (BF) (Sigma-Aldrich), recombinant Shh (r-Shh) (R&D System, USA), SAG (Enzo, USA), and cyclopamine (Sigma-Aldrich) for 1 h and then treated with the ESPs of *A*. *cantonensis* L5 [1].

### Blood-brain barrier functional assay

Evans blue infusion was employed to detect blood-brain barrier function in this study. After anesthetizing *A*. *cantonensis*-infected mice and uninfected mice with isoflurane, 2% Evans blue (Sigma-Aldrich) was intraperitoneally infused into each mouse for 1 h. The brains were then removed from the cranial cavity and fixed in 10% formalin. Blue dye was released from brain tissue extracts by N,N-dimethylformamide (Sigma-Aldrich). The OD values of the extracts were detected by spectrophotometry at 595 nm.

### SDS-PAGE electrophoresis and Western blotting analysis

Proteins from astrocytes were separated by 12% SDS-PAGE after treatment. These samples were analyzed by Western blotting; they were transferred to a nitrocellulose membrane and incubated overnight in antibodies against Shh (Sigma-Aldrich), Ptch (Sigma-Aldrich), Smo (Sigma-Aldrich), Gli-1 (Sigma-Aldrich), LC3-I (Sigma-Aldrich), LC3-II (Sigma-Aldrich), Beclin (Sigma-Aldrich), p62 (Sigma-Aldrich), and β-actin (Sigma-Aldrich). The membrane was washed three times with TBS/T for 10 min each and then incubated with a 1:10,000 dilution of horseradish peroxidase-conjugated anti-rabbit or mouse antibody (Sigma-Aldrich). The immunoreactive bands were detected by ECL reagents (EMD Millipore, USA) and captured by a UVP BioSpectrum 600 Imaging System (Upland, CA). ImageJ software analysis was used to detect the image densitometry of target proteins.

### RNA extraction and microarray analysis

Total RNA was extracted using TRIzol reagent as described in the accompanying protocol (Thermo Fisher Scientific, USA). Each total RNA sample was measured by a spectrophotometer, and its quality was checked by agarose gel electrophoresis. Labeled cDNA targets for hybridization were synthesized by reverse transcription from each total RNA sample in the presence of Cy5-dUTP and Cy3-dUTP (Amersham Pharmacia Biotech, UK). For each reverse transcription reaction, 20 μg of total RNA was mixed with 2 μg of an oligo-dT primer in a total volume of 13 μl, heated to 70°C for 10 min, and cooled on ice. To this mixture, we added 1.5 μl of 20X nucleotide cocktail (10 μM each dATP, dCTP, dGTP, and 6 μM dTTP), either 3 μl of Cy3-dUTP or Cy5-dUTP (Amersham Pharmacia Biotech), 6 μl of 5X first-strand buffer, 3 μl of 0.1 M DTT, 0.1 μl of RNAguard (Amersham Pharmacia Biotech, UK), and 2 μl of 200 units/ml Superscript II reverse transcriptase (Thermo Fisher Scientific, USA). After incubation at 42°C, the RNA strand was degraded by adding 5 μl of 0.5 N NaOH and incubating at 70°C for 10 min. The separately synthesized Cy3- and Cy5-labeled targets were combined and mixed with 20 μg of *A*. *cantonensis* DNA, and the volume was brought up to 500 μl with distilled water.

In this study, an *A*. *cantonensis* customized cDNA microarray (version 2.0) was manufactured in Molecular Regulation and Bioinformatics Laboratory, Chang Gung University. We added poly(A) RNA, SDS, formamide, and SSC (1.5 M NaCl and 150 mM sodium citrate, pH 7.0) to a concentrated target and adjusted the volume to 30 μl with distilled water. This target mixture was denatured by heating for 2 min at 100°C, incubated at room temperature for 20–30 min in a dark box, and placed on the array under a 24 mm x 40 mm glass coverslip. We incubated the arrays for hybridization at 42°C for 16 h in a humid chamber. The washed arrays were scanned using ScanArray 4000 (GSI Lumonics, UK), and the obtained data (16-bit tiff image) were analyzed by QuantArray software (GSI Lumonics, UK) by converting the signal intensity of each spot into text format. The background was subtracted using the average intensity of the blank spots. The cut-off value for each experiment was calculated as 5-9-fold of the lowest signal intensity except the blank spots, leaving approximately the top 3,000 data points with relatively high signal intensities. In each sample, the Cy3/Cy5 ratio values were log transformed, and global equalization was performed to remove the deviation of the signal intensity between whole Cy3 and whole Cy5 fluorescence by subtracting the median of all log (Cy3/Cy5) values from each log (Cy3/Cy5) value. This stipulation and the severe cut-off level allowed us to eliminate data points affected by experimental artifacts. Genes with more than three missing data values were excluded from further analyses. With each gene, variances among the samples were also calculated. The mRNA expression patterns were generated and analyzed in Heatmaps by using MultiExperiment Viewer (Mev).

### RNA extraction and Real-time qPCR

Total RNA was extracted from the astrocytes treated with *A*. *cantonensis* L5 ESPs for the indicated doses by using GENEzol TriRNA Pure Kit (Geneaid, Taiwan). The concentration of RNA was determined by with a spectrophotometer (OD260 nm). The cDNA were obtained by reverse transcription. Real-time qPCR was performed using the SYBR Green Supermix (Bio-Rad, USA) on the Real-Time PCR Detection System (Bio-Rad, USA). A GAPDH internal control was used. The expression levels were detected with specific primers: 5'-CCTCTCCTGCTATGCTCCTG-3' (sense) and 5'-GTGGCGGTTACA AAGCAAAT-3' (anti-sense) for Shh; 5'-CTCAGGCAATACGAAGCACA-3' (sense) and 5'-GACAAGGAGCCAGAG TCCAG -3' (anti-sense) for Ptch; 5'-TTAATGGTGGGAGAGGGAATGG-3' (sense) and 5'-ATCGAAGCTGTCTTCAACCC-3' (anti-sense) for Smo; 5'-GAAGGAATTCGTGTGCCATT-3' (sense) and 5'-GCAACCTTCTTGCTCACACA-3' (anti-sense) for Gli-1; 5'-GGTCCCAGCTTAGGTTCATCA-3' (sense) and 5'-TTTGCCGTGAGTGGAGTCAT-3' (anti-sense) for GAPDH.

### Transmission electron microscopy

To investigate the formation of autophagic vacuoles by transmission electron microscopy (TEM), the cells were harvested from 10 cm dishes and fixed with Karnovsky’s fixative solution in 0.2 M cacodylate buffer (pH 7.4) for 20 min at 4°C and washed three times in cacodylate buffer for 10 min. Afterwards, the cells were incubated in 1% osmium tetroxide for 2 h at 4°C and washed three times in cacodylate buffer for 10 min. Then, the cells were dehydrated through a graded ethanol series (50–100%) and embedded in Spurr embedding solution for 24 h at 70°C. The samples were cut into 70-nm ultrathin sections and stained with 1% periodic acid solution, 5% saturated uranyl acetate, and 0.4% lead citrate. These samples were observed with a JEM-1230 microscope (JEOL, Japan).

### Immunohistochemical staining

Brain tissues were collected from *A*. *cantonensis*-infected mice and fixed in 10% paraformaldehyde (PFA). The tissues were embedded in paraffin after three weeks and then sliced with an ultramicrotome. Finally, these sections were stained with hematoxylin and eosin (H&E).

### Autophagy detection

Cultured astrocytes treated with 500 μg/ml ESPs or 100 nM rapamycin were collected. Autophagy induction was detected in astrocytes by Autophagy Detection Kit 2.0 (Enzo, USA) and immunomicroscopy.

### Cell viability assay

To detect cell viability in astrocytes after treatment with ESPs or drugs, the cells (1x10^7^ cells/ml) were incubated with 50 ml of CCK-8 solution (Cell Counting Kit-8) (Sigma-Aldrich, USA) at 37°C in the dark with mild shaking for 1 h. In the presence of cells, highly water-soluble tetrazolium salt WST-8 [2-(2-methoxy-4-nitrophenyl)-3-(4-nitrophenyl)-5-(2, 4-disulfophenyl)- 2H-tetrazolium, monosodium salt] produces formazan dye. The percentage of cell viability was monitored by detecting the absorbance of the formazan dye at 450 nm using a spectrophotometer (Molecular Devices, USA).

### Statistical analysis

Student t-test and ANOVA were employed to compare the expression levels by GraphPad Prism 5 software (GraphPad, USA). The data are expressed as the mean ± standard deviation. P < 0.05 and < 0.01 was considered statistically significant.

## Results

### Histopathological findings and blood-brain barrier dysfunction in mice after *A*. *cantonensis* infection

**Fig 1:**
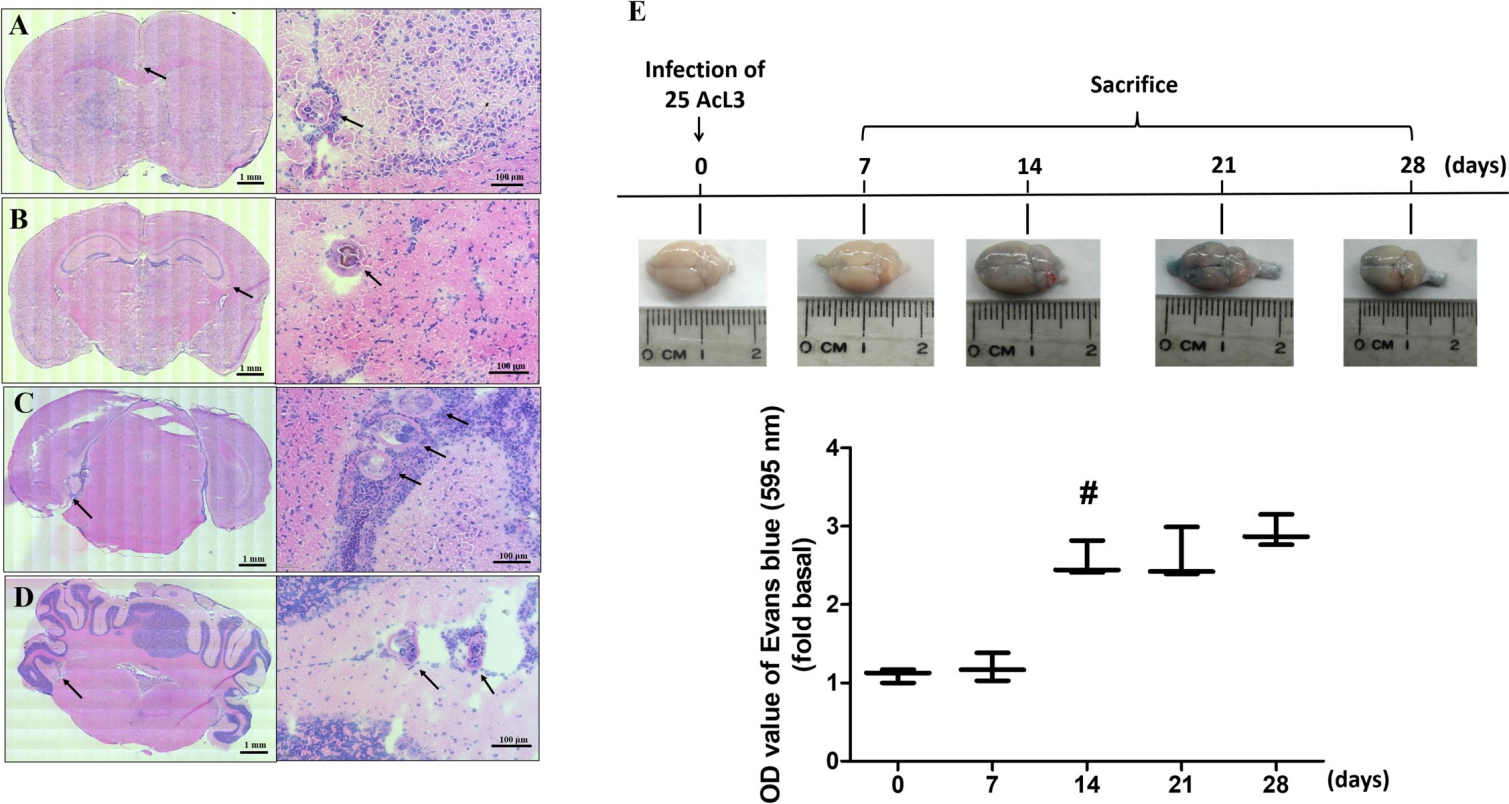
Histopathological changes and blood-brain barrier dysfunction are induced in *Angiostrongylus cantonensis-*infected mice. Fifth-stage larvae of *A*. *cantonensis* were observed in the (A) anterior cerebral fissure, (B) hippocampus, (C) posterior cerebral fissure, and (D) cerebellar fissure of mice infected with 25 third-stage larvae on day 14 postinfection (stained with hematoxylin-eosin). Inflammatory cells were found surrounding these larvae. (E) Blood-brain barrier dysfunction and breakdown was shown by positive staining after the intravenous infusion of 2% Evans blue into the brains of mice infected with *Angiostrongylus cantonensis* on days 0, 7, 14, 21 and 28 postinfection.

To investigate the effect of *A*. *cantonensis* infection in brain tissue, nonpermissive hosts (mice) were infected with 25 third-stage larvae (L3) by stomach intubation. By staining with hematoxylin and eosin, the fifth-stage larvae (L5) of *A*. *cantonensis* were observed in brain sections from infected mice on day 14 postinfection. These larvae were found in the anterior cerebral fissure (Fig 1A), hippocampus (Fig 1B), posterior cerebral fissure (Fig 1C), and cerebellar fissure (Fig 1D). In addition, the number of inflammatory cells surround these larvae was significantly increased.

The blood-brain barrier (BBB) is formed by astrocytes and endothelial cells and regulates the transport of molecules, circulating blood, and pathogens into the central nervous system (CNS) [34]. To investigate the function of the BBB after *A*. *cantonensis* infection, Evans blue staining was utilized to detect changes in BBB integrity. This dye is permeable to the BBB and stained brain tissues. After intravenous infusion, Evans blue staining was significantly elevated in *A*. *cantonensis*-infected mouse brains on days 7, 14, and 28 postinfection (Fig 1E). However, mouse brains without infection showed a normal appearance. These findings indicate BBB dysfunction and breakdown were induced in mouse brains after *A*. *cantonensis* infection.

### ESPs induce the expression of autophagy-related genes in astrocytes

**Fig 2:**
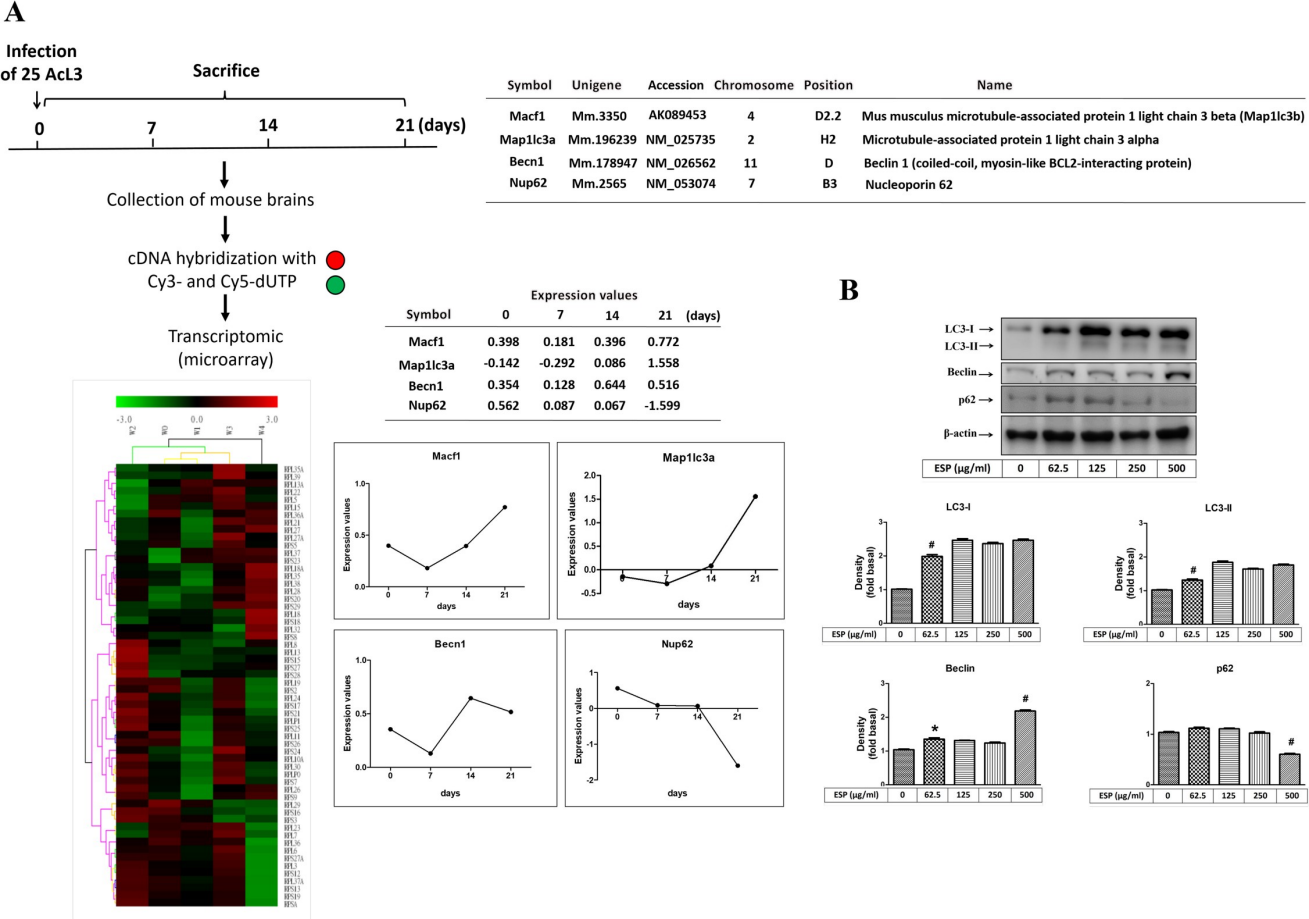
Excretory/secretory products of *A*. *cantonensis* L5 stimulate autophagy in astrocytes. (A) Mouse brains were collected from *A*. *cantonensis*-infected mice on days 0, 7, 14, and 21 postinfection. Then, the mRNA expression levels of autophagy-related molecules were detected by cDNA microarray analysis. (B) Cells were treated with 0, 62.5, 125, 250, and 500 μg/ml *A*. *cantonensis* L5 excretory/secretory products (ESPs) for 12 h. The protein expression levels of autophagy-related molecules were detected by Western blotting. The data are expressed as the means ± SD from three independent experiments (*n* = 3). *P<0.05, ^#^*P*<0.01, compared with the respective values of cells treated with 0 μg/ml *A*. *cantonensis* L5 ESPs for 12 h.

To investigate autophagy induction in mouse brains after *A*. *cantonensis* infection, a cDNA microarray was utilized to monitor the expression of autophagy-related genes, such as LC3B (Macf1 and Map1lc3a), Beclin 1 (Becn1), and p62 (Nup62). The microarray data showed that the expression levels of LC3B and Beclin 1 were elevated in *A*. *cantonensis*-infected mouse brains on day 14 and 21 postinfection but that the expression of p62 was decreased from days 7 to 21 postinfection (Fig 2A). Moreover, the Western blotting data showed that the protein expression levels of the autophagy-related proteins LC3-I, LC3-II, Beclin, and p62 were dose-dependently elevated in astrocytes upon treatment with the excretory/secretory products (ESPs) of *A*. *cantonensis* L5 (Fig 2B). These results suggest that *A*. *cantonensis* L5 induces autophagy via the secretion of ESPs in astrocytes.

### ESPs induce autophagosome formation

**Fig 3:**
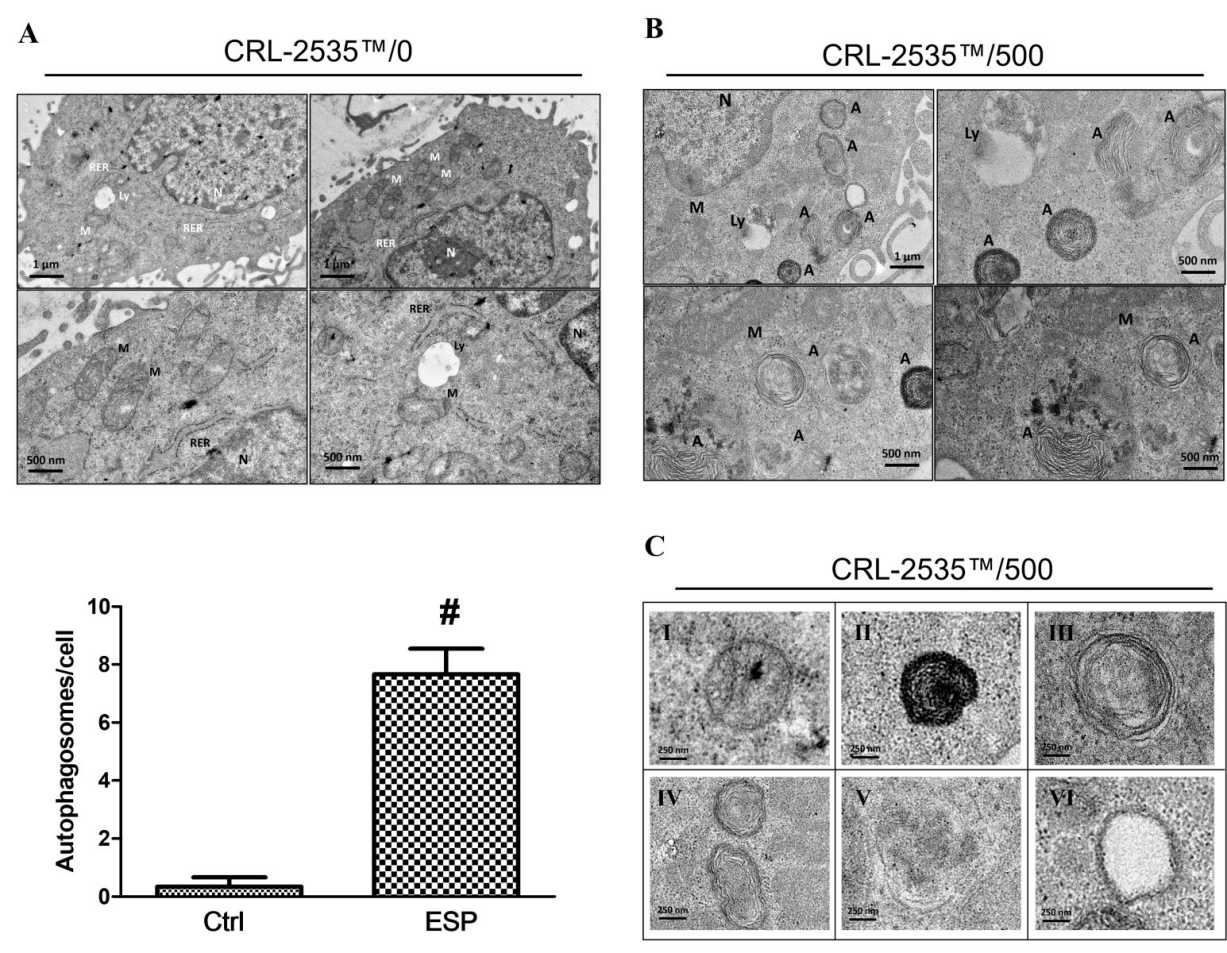
Excretory/secretory products of *A*. *cantonensis* L5 stimulate autophagic-like vacuole formation. Cells were (A) left untreated or (B) treated with 500 μg/ml *A*. *cantonensis* L5 excretory/secretory products (ESPs) for 12 h. The formation of autophagic-like vacuoles was detected by using transmission electron microscopy (TEM) (A: autophagosomes. M: mitochondria. Ly: lysosomes. RER: rough endoplasmic reticulum. N: nucleus). (C) TEM images of autophagic structures upon ESPs treatment ((I) (II) autolysosomes with dark undigested content; (III) autophagosomes; (IV) autolysosomes filled with undigested lipids; (V) phagophores; and (VI) empty autophagic-like vacuoles).

Transmission electron microscopy (TEM) is commonly utilized to investigate autophagy induction. TEM can be utilized to visualize autophagy in different forms, such as autolysosomes, autophagosomes, and other autophagic-like vacuoles, in astrocytes. As shown in Fig 3, electron microscopy analysis of astrocytes (CRL-2535) treated with 500 μg/ml *A*. *cantonensis* L5 ESPs revealed that the number of autophagosome-like structures was significantly increased in the cytoplasm compared to that in the control group (Fig 3A and 3B). Moreover, the detailed TEM data showed that autophagy-related vacuoles of different stages, including autolysosomes, autophagosomes, phagophores, and empty autophagic-like vacuoles, were observed upon 500 μg/ml *A*. *cantonensis* L5 ESPs treatment (Fig 3C).

### Rapamycin and ESPs induce autophagy in astrocytes

**Fig 4:**
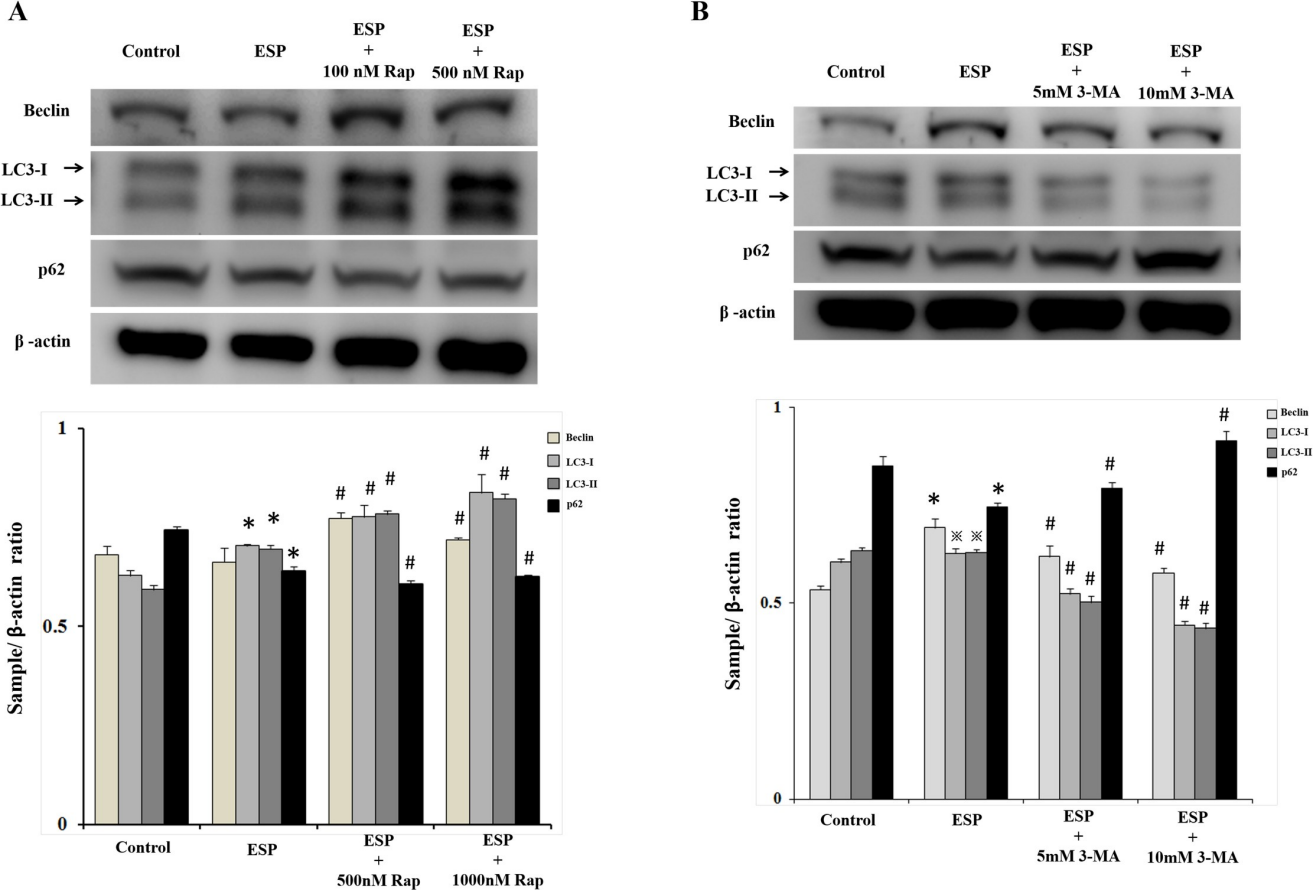
Rapamycin induces autophagy in excretory/secretory product-treated astrocytes. (A) Cells were pretreated with 100 or 500 nM rapamycin for 1 h and then incubated with 500 μg/ml *A*. *cantonensis* L5 excretory/secretory products (ESPs) for 12 h. Western blotting was used to analyze the expression levels of the autophagy-related proteins LC3-I, LC3-II, Beclin, and p62. β-actin is shown as the control. (B) Cells were pretreated with 5 or 10 mM 3-methyladenine (3-MA) for 1 h and then incubated with 500 μg/ml *A*. *cantonensis* L5 excretory/secretory products (ESPs) for 12 h. Western blotting was used to analyze the expression levels of the autophagy-related proteins LC3-I, LC3-II, Beclin, and p62. β-actin is shown as the control. The data are expressed as the means ± SD from three independent experiments (*n* = 3). ^※^*P*<0.05, *P<0.01, compared with the respective values of the control. ^#^*P*<0.01, compared with the cells exposed to ESPs.

**Fig 5:**
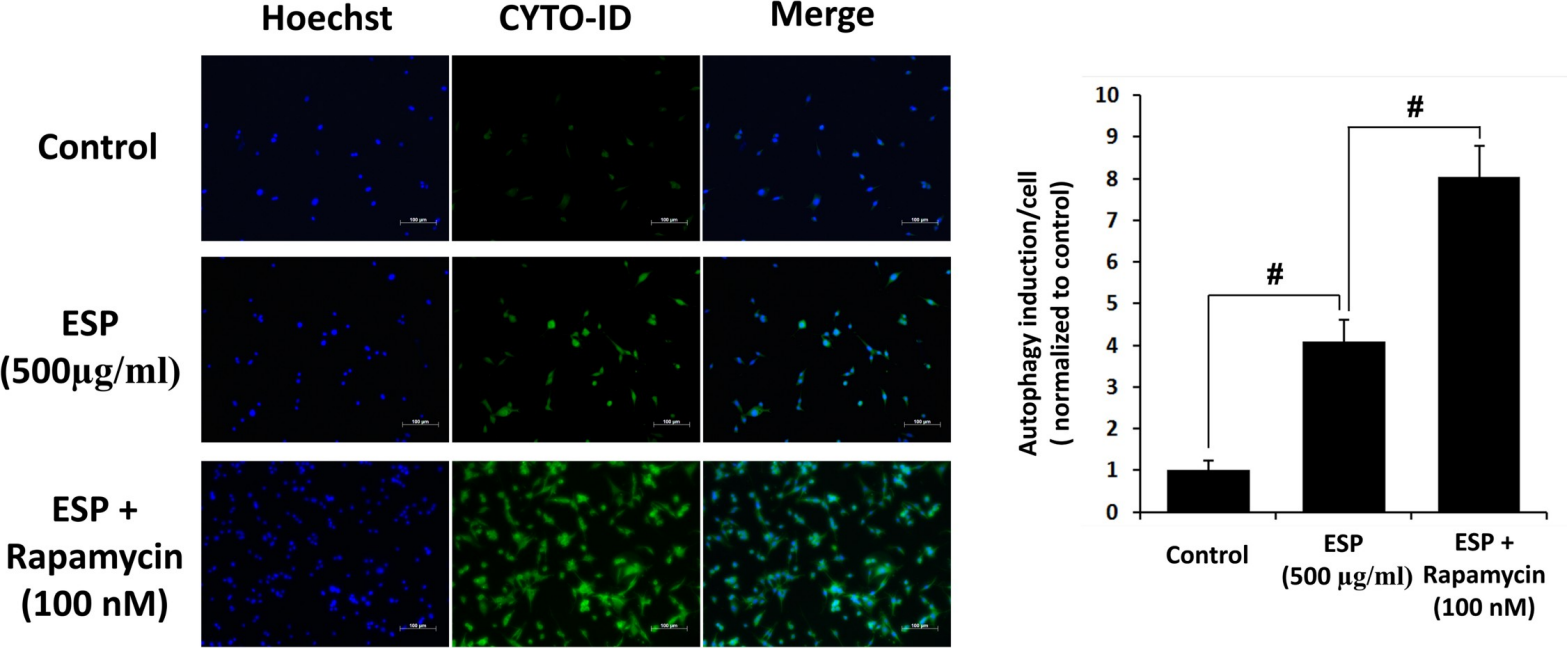
Immunofluorescence staining of autophagy-related proteins in excretory/secretory product-treated astrocytes. Cells were pretreated with 100 nM rapamycin for 1 h and then incubated with 500 μg/ml of *A*. *cantonensis* L5 excretory/secretory products (ESPs) for 12 h. Immunofluorescence staining was used to detect the expression of autophagy-related proteins (blue: nucleus; green: autophagy).

To determine the effects of autophagy induction, rapamycin, a specific autophagy activator, was employed to activate the autophagy-related pathway. Rapamycin can bind to the FK506-binding protein (FKBP12) and then form a complex. Ultimately, this complex inactivates mTOR and induces autophagosome formation [35,36]. However, the effects of rapamycin on nematode-induced autophagy are still unclear. Astrocytes were pretreated with different concentrations of rapamycin (100 and 500 nM) and then incubated with ESPs for 12 h. Western blotting was used to detect the protein expression levels of the autophagy-related proteins LC3-I, LC3-II, Beclin, and p62 (Fig 4A). The results showed that the expression levels of LC3-I, LC3-II, and Beclin were significantly increased in the ESPs alone-treated group compared to the control group, and the expression of p62 was significantly decreased. On the other hand, rapamycin influenced the expression of LC3-I, LC3-II, Beclin, and p62 in the rapamycin- and ESPs-treated groups compared to the ESPs alone-alone group. Finally, we used the autophagy inhibitor 3-methyladenine (3-MA) to observe autophagy induct upon ESPs treatment. The data showed that 3-MA reduced the protein expression levels of autophagy-related proteins upon ESPs treatment (Fig 4B). Double immunofluorescence staining for nuclei and autophagic vacuoles showed that the number of autophagosome-like vacuoles was significantly increased in the rapamycin- and ESPs-treated groups compared to the control and ESPs alone-treated groups (Fig 5). These results suggest that rapamycin and ESPs can activate the mechanism of autophagy induction.

### Protective effect of autophagy against ESPs-induced cell death in astrocytes

**Fig 6:**
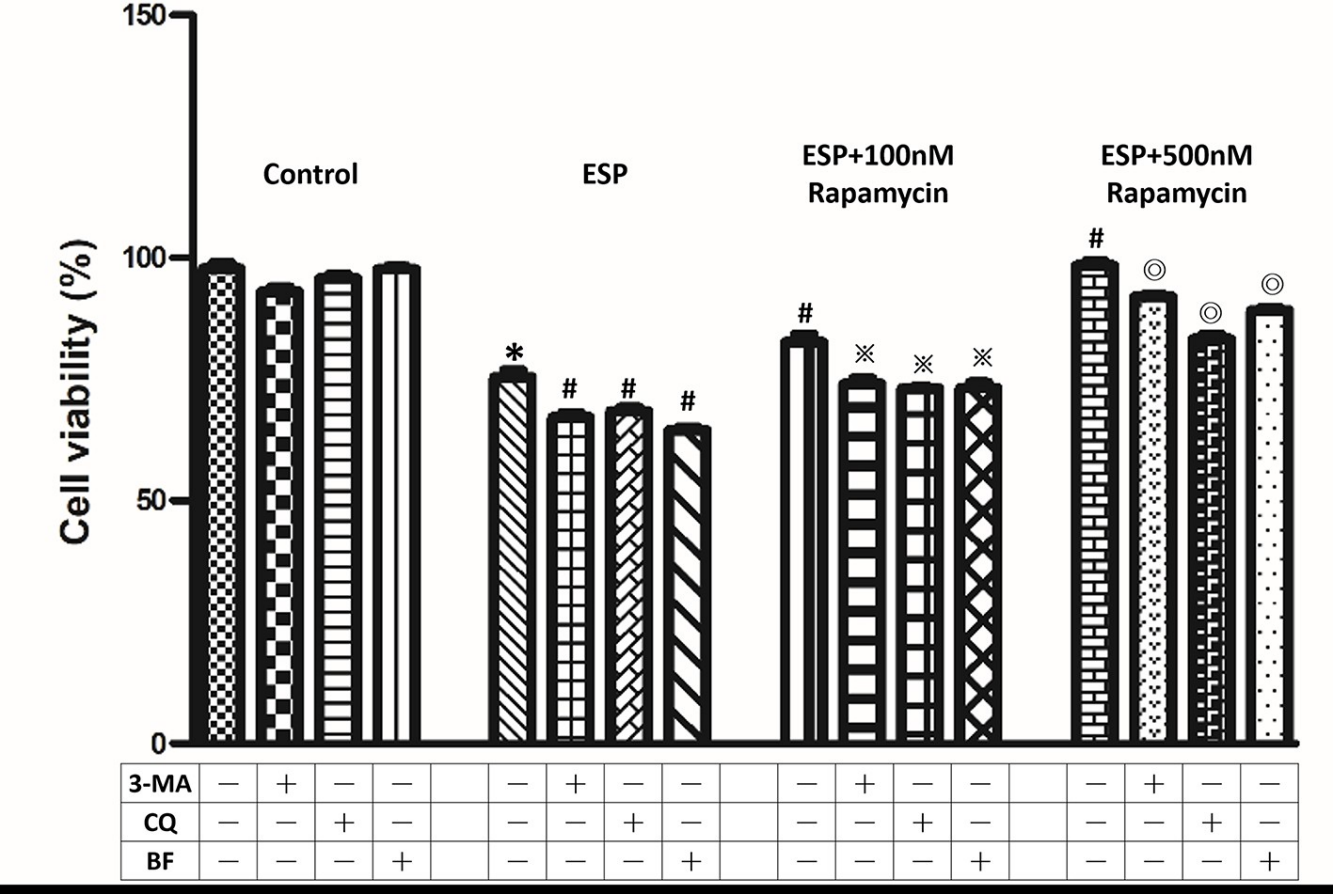
Rapamycin protects astrocytes upon excretory/secretory product treatment via autophagy induction. Cells were pretreated with rapamycin, 3-methyladenine (3-MA), chloroquine (CQ), or bafilomycin A1 (BF) for 1 h and then incubated with 500 μg/ml *A*. *cantonensis* L5 excretory/secretory products (ESPs) for 12 h. The viability of astrocytes was analyzed by the CCK-8 assay. The data are expressed as the means ± SD from three independent experiments (*n* = 3). *P<0.01, compared with the control. ^#^*P*<0.01, compared with the cells exposed to ESPs. ^※^*P*<0.01, compared with the cells exposed to ESPs+100 nM rapamycin. ^◎^*P*<0.01, compared with the cells exposed to ESPs+500 nM rapamycin.

To investigate the protective effects of autophagy in ESPs-treated astrocytes, cells were pretreated with an autophagy activator or inhibitor and then incubated with ESPs for 12 h (Fig 6). First, we wanted to determine the effect of ESPs in astrocytes. Cells were pretreated with or without 500 μg/ml *A*. *cantonensis* L5 ESPs and then cell viability was observed by the CCK8 assay. The data showed that ESPs preparations decreased the viability of astrocytes. Furthermore, we determined whether autophagy activation can protect astrocytes upon *A*. *cantonensis* L5 ESPs treatment. Cells were pretreated with an autophagy activator (rapamycin) or inhibitor (3-methyladenine (3-MA), chloroquine (CQ), and bafilomycin A1 (BF)). First, the data showed that 3-MA, CQ, and BF reduced cell viability by inactivating the autophagy-related pathway in ESPs-treated astrocytes. Conversely, the percentage of cell viability was dose-dependently significantly increased following the activation of the autophagy-related pathway by rapamycin. These results demonstrate that autophagy induction increases the viability of ESPs-treated astrocytes.

### ESPs induce Shh signaling pathway activation

**Fig 7:**
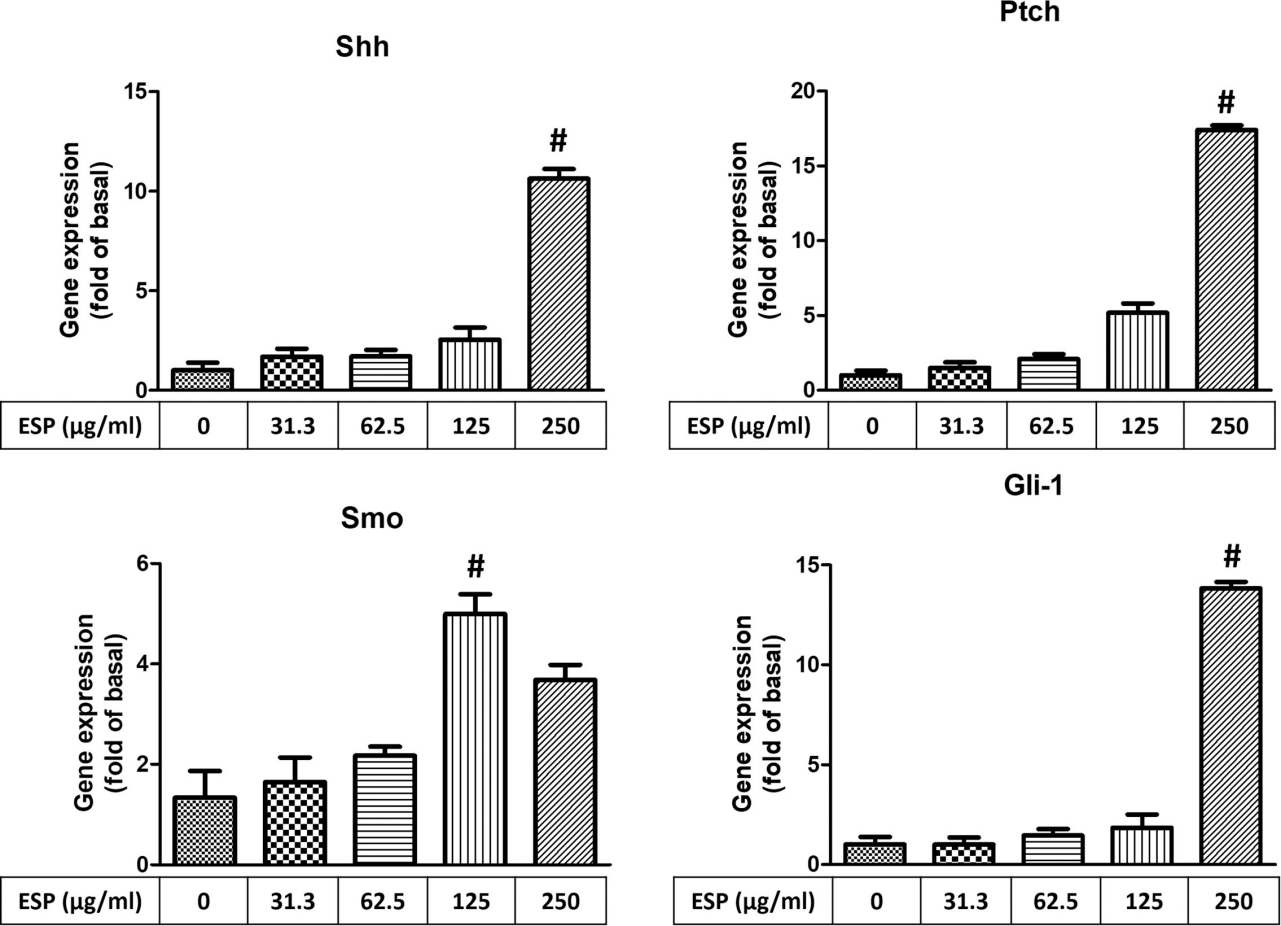
Excretory/secretory products induce gene expressions of Shh signaling pathway. Cells were treated with 0, 31.3, 62.5, 125, and 250 μg/ml *A*. *cantonensis* L5 excretory/secretory products (ESPs) for 12 h. The mRNA expression levels of Shh signaling pathway-related molecules (Shh, Ptch, Smo, and Gli-1) were detected by Real-Time qPCR. The data are expressed as the means ± SD from three independent experiments (*n* = 3). ^#^*P*<0.01, compared with the respective values of cells treated with 0 μg/ml *A*. *cantonensis* L5 ESPs for 12 h.

**Fig 8:**
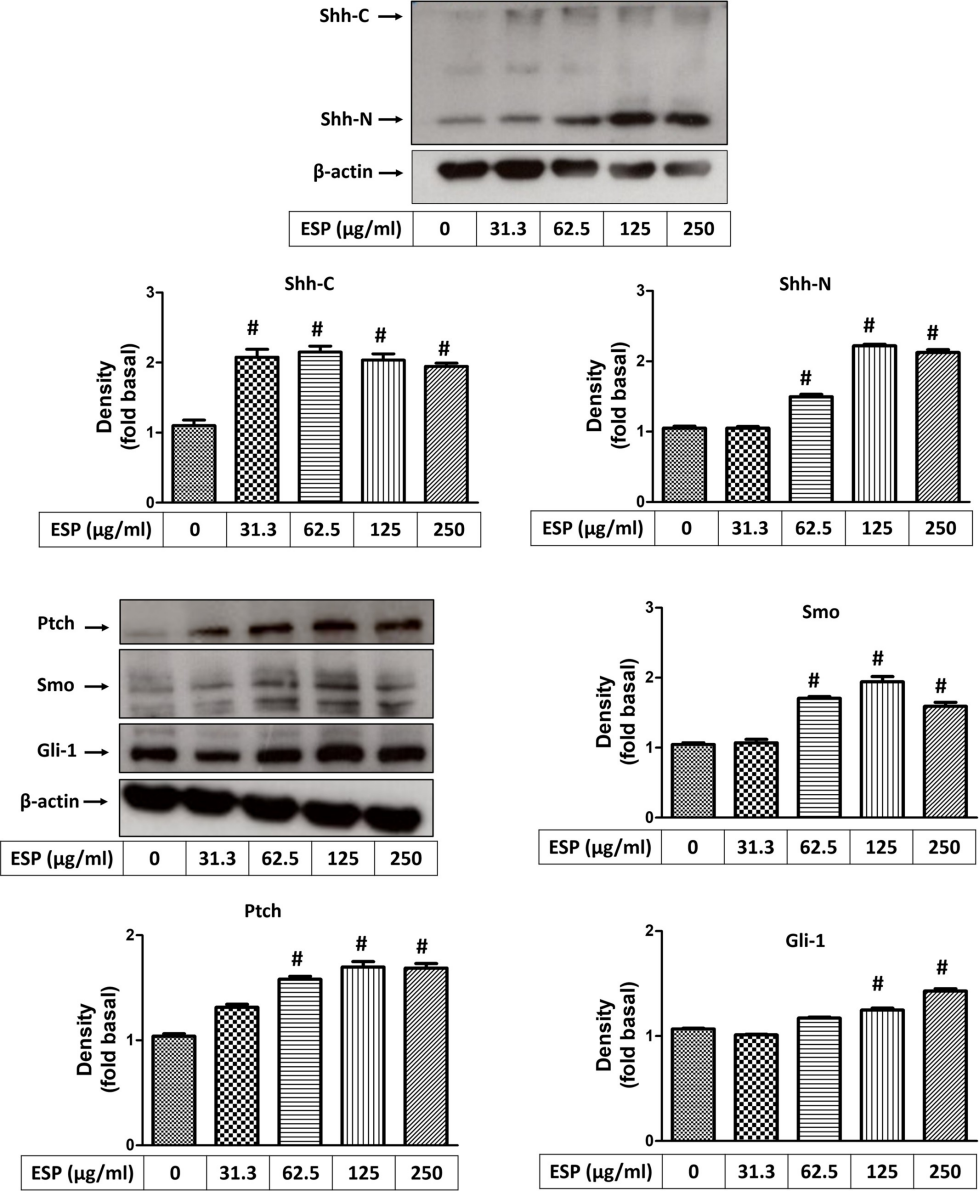
Excretory/secretory products induce protein expressions of Shh signaling pathway. Cells were treated with 0, 31.3, 62.5, 125, and 250 μg/ml *A*. *cantonensis* L5 excretory/secretory products (ESPs) for 12 h. The protein expression levels of Shh signaling pathway-related molecules (Shh-N, Shh-C, Ptch, Smo, and Gli-1) were detected by Western blotting. The data are expressed as the means ± SD from three independent experiments (*n* = 3). ^#^*P*<0.01, compared with the respective values of cells treated with 0 μg/ml *A*. *cantonensis* L5 ESPs for 12 h.

To determine whether the Shh signaling pathway is activated in astrocytes after *A*. *cantonensis* L5 ESPs treatment, Real-Time qPCR and Western blotting analysis were used to determine the expression of downstream molecules of the Shh pathway. The Real-Time qPCR data showed that the mRNA expression levels of Shh pathway-related molecules were significantly increased upon ESPs treatment (Fig 7). On the other hands, the Western blotting results showed that Shh-N (functional domain) and Shh-C (catalytic domain) were significantly elevated upon treatment with 62.5 and 31.3 μg/ml ESPs and that Ptch, Smo, and Gli-1 were significantly elevated upon treatment with 62.5 μg/ml ESPs (Fig 8). These results confirm that ESPs activate the Shh signaling pathway in astrocytes.

### ESPs induce autophagy-related protein expression through the Shh signaling pathway

**Fig 9:**
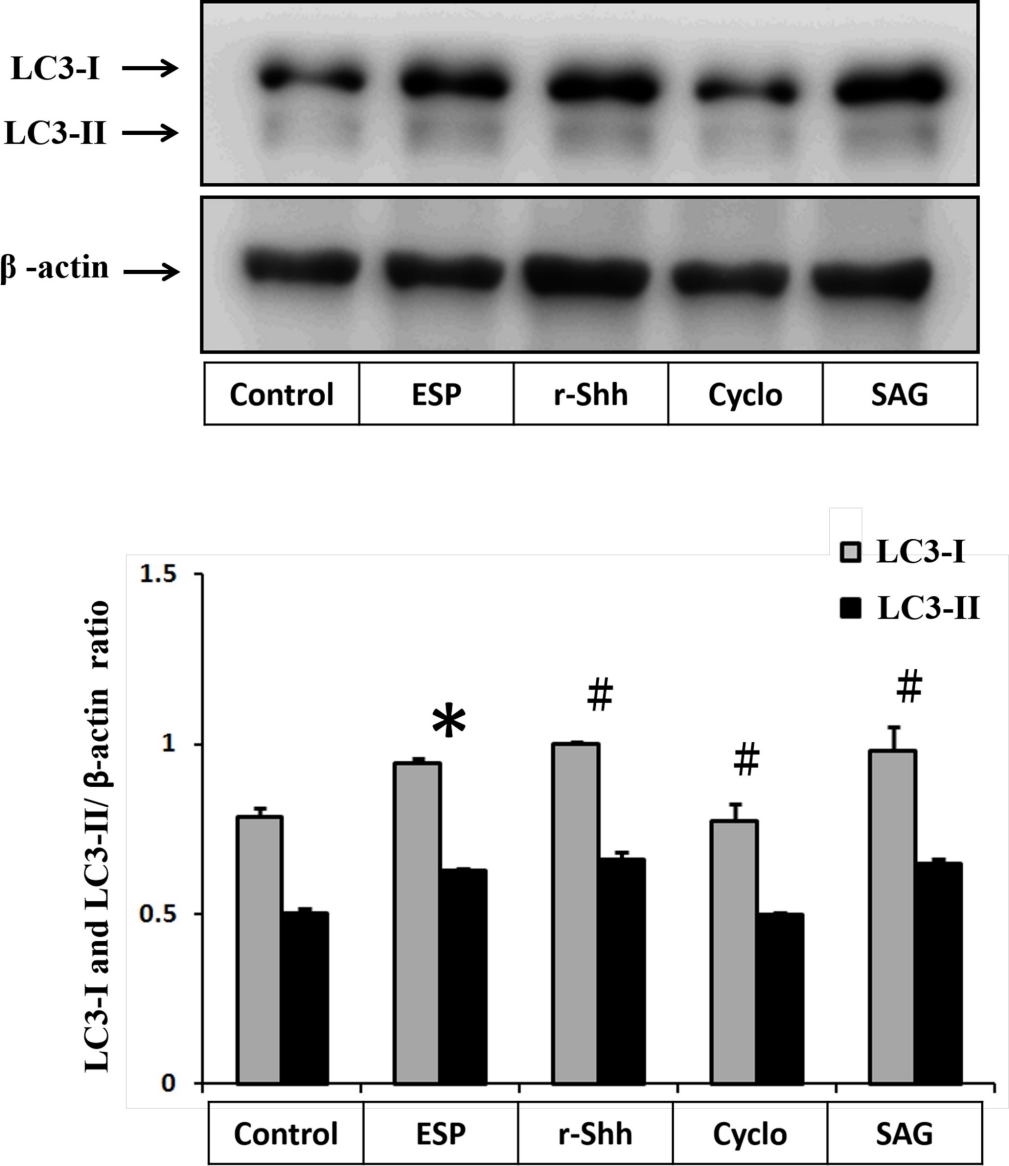
Excretory/secretory products induce autophagy through the Shh signaling pathway. Cells were pretreated with recombinant Shh (r-Shh), Shh agonist (SAG), and cyclopamine (Cyclo) for 1 h and then incubated with 500 μg/ml *A*. *cantonensis* L5 excretory/secretory products (ESPs) for 12 h. The protein expression levels of LC3-I and LC3-II were detected by Western blotting. The data are expressed as the means ± SD from three independent experiments (*n* = 3). *P<0.01, compared with the control. ^#^*p*<0.01, compared with the cells exposed to ESPs.

To determine whether Shh signaling can stimulate autophagy induction in ESPs-treated astrocytes, cells were pretreated with recombinant Shh (r-Shh), a Shh agonist (SAG), and a Shh pathway inhibitor (cyclopamine) and then treated with *A*. *cantonensis* L5 ESPs. Western blotting analysis was employed to detect the protein expression of autophagy-related molecules. The data showed that r-Shh and SAG stimulated LC3-I and LC3-II expression (Fig 9) by activating the Shh pathway in ESPs-treated astrocytes. Conversely, the expression levels of LC3-I and LC3-II were significantly decreased following inactivation of the Shh pathway by cyclopamine. These results suggest that the ESPs stimulate autophagy induction through the Shh signaling pathway in astrocytes.

## Discussion

During parasitic helminth infection, parasites, including *Angiostrongylus cantonensis*, *Ancylostoma caninum*, hookworm, *Trichinella spiralis*, *Trichuris suis*, *Haemonchus contortus*, *Anisakis pegreffii* and *Contracaecum osculatum*, can produce excretory-secretory products (ESPs) to modulate the host response [33,37–43]. ESPs play an important role in the interaction between parasites and hosts and are essential factors for host tissue invasion, digestion, feeding, development, molting, host immune responses, oxidative stress, and apoptosis [40,44]. The ESPs of nematodes contain many molecules, including proteins, carbohydrates, and fatty acids [45,46]. In studies of the immunomodulatory function of ESPs, helminths have been shown produce ESPs to induce the generation of Th2 cytokine (IL-4, IL-10, and IL-13) by the host, inflammation, and the elevation of IgE and eosinophilia levels in the serum [47]. They can also drive antigen-presenting cells (dendritic cells) to switch from a Th1-mediated response to the Th2 phenotype by reducing IFN-γ production [48–50]. In our previous study, we employed proteomic and bioinformatics analyses to determine the component molecules in *A*. *cantonensis* L5 ESPs. These results showed that approximately 51 protein spots were identified, and immunoreactive proteins including disulfide isomerases and aspartic proteases were highly expressed. These two proteins play important roles in invasion, digestion, and host immune modulation [33].

Humans are nonpermissive hosts (accidental hosts) for *A*. *cantonensis*. In *A*. *cantonensis* infection, worms usually cause severe damage and immune responses, such as eosinophilic meningitis and meningoencephalitis, in the central nervous system in humans. In our previous study, we employed immunohistochemistry to analyze the expression of cytokines in mouse brains. The results showed that the expression levels of Th-2-related cytokines (IL-4, IL-10, and IL-13) are increased in various regions of the brain, such as the isocortex, olfactory area, hippocampus, thalamus, and cerebellar nuclei [50]. Moreover, hematoxylin and eosin were used to examine pathological changes in the brains of *A*. *cantonensis-*infected mice. Eosinophilic meningitis, encephalitis, perivascular cuffing, hemorrhage and meninges thickness were observed in the infected brain tissue, but the detailed mechanism is still not clearly understood [51]. In the present studies, we found fifth-stage larvae (L5) of *A*. *cantonensis* and eosinophils in various regions of the brain.

The infection of the brain with parasites is a complicated procedure that requires closed parasite-host interactions. During infection, parasitic worms require transmission to the central nervous system (CNS) through the blood-brain barrier (BBB), and they can secrete several molecules to promote their penetration into the brain parenchyma through the BBB [52]. In this study, we used Evans blue dye to detect the function of the BBB in mice. The data showed that BBB breakdown was induced after *A*. *cantonensis* infection. The BBB is formed by astrocytes, endothelial cells, and pericytes. This barrier separates blood vessels and brain tissues, and it only allows small molecules, including O_2_, CO_2,_ hormones, and glucose, to infiltrate the CNS [53]. Therefore, the BBB can protect against toxin and pathogen infection, including parasitic infection, in the CNS. Previous studies have demonstrated that only 2% of small molecule drugs can cross the BBB and infiltrate the CNS [54]. Many studies have revealed that, during parasitic infection, parasites (protozoans and helminths), such as *Trypanosoma evansi*, Leishmania, *Plasmodium falciparum*, *Taenia solium*, and *Toxoplasma gondii*, infiltrate the CNS and induce brain damage via BBB breakdown [55–59]. Research on *A*. *cantonensis* has also demonstrated that BBB disruption occurs in mice after *A*. *cantonensis* infection [60].

Astrocytes are the most abundant glial cells in the mammalian brain. They can regulate neural cell differentiation and brain homeostasis by secreting neural transmitters. During infection with microbes, such as bacteria [61], viruses [62], and protozoa [63–65], astrocytes also play an important role in pathogen clearance via the stimulation of host immune responses in the CNS. Although astrocytes are an important source of factors that trigger the mechanism of pathogen clearance, their pathway has still not been identified in helminth infections. Our study found that *A*. *cantonensis* L5 ESPs induce oxidative stress, cell apoptosis, and cytokine secretion in astrocytes [1,9]. The data demonstrate that afterwards, *A*. *cantonensis* L5 ESPs stimulate the expression of autophagy-related proteins and induce autophagosome formation in astrocytes. Moreover, autophagy induction can protect astrocytes upon treatment with *A*. *cantonensis* L5 ESPs. Many studies have demonstrated that autophagy is induced in astrocytes exposed to different conditions, such as Alzheimer’s disease, ischemia, oxygen-glucose deprivation, nutrient deprivation, ethanol toxicity, and neurosteroid treatment [66–71]. Autophagy can also be activated during pathogen infection. Many viruses have developed protective abilities by antagonizing pathogen clearance mechanisms [72,73].

In this study, we found that *A*. *cantonensis* L5 ESPs induce autophagy generation and that Sonic hedgehog (Shh) plays an important role in activating this mechanism. In our previous study, we found that *A*. *cantonensis* L5 ESPs induce the activation of the Shh signaling pathway by increasing the expression of Shh signaling-related molecules (Shh, Ptch-1 and Gli-1). However, cell apoptosis in astrocytes is significantly decreased after Shh signaling pathway activation [1]. A previous study demonstrated that autophagy induction can alleviate apoptosis in astrocytes [74,75]. In conclusion, the present study confirmed that *A*. *cantonensis* L5 ESPs can induce autophagy in mouse astrocytes. Moreover, the Shh signaling pathway plays an important role in increasing cell viability by stimulating the expression and activity of autophagy upon treatment with *A*. *cantonensis* L5 ESPs and rapamycin. In conclusion, this study we research on the molecular mechanisms of autophagy in astrocytes after *A*. *cantonensis* L5 ESPs treatment. We found that Shh signaling pathway plays a protective role for astrocytes through induction of autophagy. It may be a good target to develop the new therapy for Angiostrongyliasis.
